# Impact evaluation of a maternal and neonatal health training intervention in private Ugandan facilities

**DOI:** 10.1093/heapol/czab072

**Published:** 2021-06-29

**Authors:** Joy Noel Baumgartner, Jennifer Headley, Julius Kirya, Josh Guenther, James Kaggwa, Min Kyung Kim, Luke Aldridge, Stefanie Weiland, Joseph Egger

**Affiliations:** School of Social Work, University of North Carolina, 325 Pittsboro Street, Chapel Hill, NC 27599-3550, USA; Duke Global Health Institute, Duke University, 310 Trent Dr, Durham, NC 27710, USA; Duke Global Health Institute, Duke University, 310 Trent Dr, Durham, NC 27710, USA; LifeNet International, 64-25 Ring Road, Muyenga PO Box 21189, Kampala, Uganda; LifeNet International, 64-25 Ring Road, Muyenga PO Box 21189, Kampala, Uganda; LifeNet International, 64-25 Ring Road, Muyenga PO Box 21189, Kampala, Uganda; Harvard T. H. Chan School of Public Health, 677 Huntington Ave, Boston, MA 02115, USA; Johns Hopkins Bloomberg School of Public Health, 615 N Wolfe St, Baltimore, MD 21205, USA; American Leprosy Missions, Greenville, SC 29601, USA; Duke Global Health Institute, Duke University, 310 Trent Dr, Durham, NC 27710, USA

**Keywords:** maternal health, neonatal health, private sector, clinical adherence, quality of care

## Abstract

Global and country-specific targets for reductions in maternal and neonatal mortality in low-resource settings will not be achieved without improvements in the quality of care for optimal facility-based obstetric and newborn care. This global call includes the private sector, which is increasingly serving low-resource pregnant women. The primary aim of this study was to estimate the impact of a clinical and management-training programme delivered by a non-governmental organization [LifeNet International] that partners with clinics on adherence to global standards of clinical quality during labour and delivery in rural Uganda. The secondary aim included describing the effect of the LifeNet training on pre-discharge neonatal and maternal mortality. The LifeNet programme delivered maternal and neonatal clinical trainings over a 10-month period in 2017–18. Direct clinical observations of obstetric deliveries were conducted at baseline (*n* = 263 pre-intervention) and endline (*n* = 321 post-intervention) for six faith-based, not-for-profit primary healthcare facilities in the greater Masaka area of Uganda. Direct observation comprised the entire delivery process, from initial client assessment to discharge, and included emergency management (e.g. postpartum haemorrhage and neonatal resuscitation). Data were supplemented by daily facility-based assessments of infrastructure during the study periods. Results showed positive and clinically meaningful increases in observed handwashing, observed delayed cord clamping, partograph use documentation and observed 1- and/or 5-minute APGAR assessments (rapid scoring system for assessing clinical status of newborn), in particular, between baseline and endline. High-quality intrapartum facility-based care is critical for reducing maternal and early neonatal mortality, and this evaluation of the LifeNet intervention indicates that their clinical training programme improved the practice of quality maternal and neonatal healthcare at all six primary care clinics in Uganda, at least over a relatively short-term period. However, for several of these quality indicators, the adherence rates, although improved, were still far from 100% and could benefit from further improvement via refresher trainings and/or a closer examination of the barriers to adherence.

**Key messages**

- The LifeNet International training intervention improved the majority of the pre-specified maternal and neonatal quality of care (QoC) indicators at rural primary care clinics in Uganda.
- However, there are still pronounced clinical quality gaps that should be addressed, including routine provider handwashing, maintaining sterility of gloves prior to use, timely use of the partograph, APGAR assessment and delayed cord clamping.
- Direct observations of clinical practice are essential for monitoring QoC and highlighting areas for improvement.

## Introduction

Global and country-specific targets for reductions in maternal and neonatal mortality in low-resource settings will not be achieved without significant improvements in optimal facility-based obstetric and newborn care (Koblinsky *et al.*, 2016; Lawn *et al.*, 2014; Campbell *et al.*, 2016). In Uganda, the maternal mortality ratio is 336 maternal deaths per 100 000 live births, and the neonatal mortality rate is 27 deaths within the first month of life per 1000 live births (Uganda Bureau of Statistics (UBOS) and ICF, 2018). These statistics represent only modest gains over the last decade (DHS, 2016). While rates of facility-based deliveries have increased in Uganda and the proportion of those deliveries attended by skilled providers has also increased, mortality data indicate that progress on maternal and neonatal health (MNH) outcomes has been stagnant (Uganda Bureau of Statistics (UBOS) and Macro International Inc., 2007; Uganda Bureau of Statistics (UBOS) and ICF International Inc., 2012; Uganda Bureau of Statistics (UBOS) and ICF, 2018; Republic of Uganda, 2016).

Both the Every Newborn Action Plan and the World Health Organization (WHO) Strategy for Ending Preventable Maternal Mortality prioritize actions to improve quality of care (QoC) at birth, going beyond simply increasing coverage (WHO, 2015a; 2014a). There is increasing global attention to closing the gap between coverage and QoC for facility-based deliveries. The WHO framework for Maternal, Newborn and Child Health QoC highlights that evidenced-based clinical care is only one of eight domains of QoC that must be addressed in comprehensive high-quality health services (Tunçalp *et al.*, 2015). There are also calls for developing better and more standardized measures of QoC for obstetric and newborn care with wide recognition that direct clinical observations (DCOs) are still the gold standard for monitoring adherence to clinical evidence-based best practices (WHO, 2014b; 2016; 2018; Semrau *et al.*, 2017).

The evidence to support effective QoC interventions addressing maternal and neonatal mortality in low-resource settings via strengthened facility-based intrapartum care is thus far mixed. A review of strategies for improving provider performance revealed that multi-faceted approaches such as training plus strengthen infrastructure and management support were more likely to have a stronger evidence base (Rowe *et al.*, 2018). However, often findings indicate that changes in essential birth practices do not have an impact on health outcomes such as the Better Birth Trial in India (Semrau *et al.*, 2017; Kara *et al.*, 2017). While that trial showed that providers were implementing components of the WHO Safe Childbirth Checklist, there was no significant effect on maternal or perinatal mortality. In Uganda and Zambia, a multi-donor, health systems initiative was designed to reduce deaths related to pregnancy and childbirth called ‘Saving Mothers, Giving Life’. Results revealed that while they documented a significant reduction in facility-based maternal mortality, they found no significant change in pre-discharge neonatal mortality rates and no significant differences in women’s reports of receiving evidence-based clinical services during delivery (Conlon *et al.*, 2019; Kruk *et al.*, 2016). The authors highlight that more direct observations and refined measurement of labour and delivery processes could have improved their QoC assessments.

Compounding the QoC evidence gap is that many evaluations focus exclusively on public sector services (Evans *et al.*, 2018) yet, increasingly, the private sector is serving low-resource pregnant women and is a critical component of national health systems (McPake and Hanson, 2016). In Uganda, ∼50% of health services are provided by faith-based organizations (Olivier *et al.*, 2015), and 22% of Ugandan women give birth in private sector health facilities with wide variation by region (DHS, 2016). While urban areas have more women with higher socioeconomic status accessing private facilities, in some rural areas, the closest health facility is a faith-based private health facility (DHS, 2016). Clinical social franchising is a growing private sector intervention in low-resource settings that purports to increase the utilization of quality services yet we cannot assume that the private sector is offering higher technical quality in the absence of evidence (Montagu *et al.*, 2016; Hirose *et al.*, 2018).

The primary aim of this study was to estimate the impact of LifeNet International’s clinical and management-training programme on adherence to global standards of clinical quality during labour and delivery by health providers in rural, private sector health facilities in the greater Masaka region of Uganda. Secondary aims included describing the effect of LifeNet’s clinical training modules on pre-discharge neonatal mortality and maternal mortality. A detailed analysis of QoC practices can better highlight the challenges and opportunities for strengthening high-quality obstetric and newborn care and its potential impact on maternal and neonatal outcomes in low-resource settings.

## Materials and methods

This quasi-experimental study was a pretest–posttest observational study design to estimate the effects of a clinical and management training intervention delivered by an international non-governmental organization (NGO) [LifeNet International] on improvements in QoC for maternal and neonatal healthcare in six LifeNet-affiliated health facilities in the greater Masaka district area, Uganda. The training intervention period was August 1, 2017 to May 29, 2018. Baseline data were collected prior to initiation of the LifeNet training programme (May 15 to July 17, 2017) and post-intervention endline data were collected immediately after the completion of the training programme (May 29 to August 12, 2018).

### Intervention

LifeNet International, a registered as a 501(c)(3) nonprofit organization in the USA, and an international NGO in Uganda, had previously developed and implemented an integrated health training package that requires 2-years of engagement with selected partner facilities in Uganda. For the purposes of this study, a modified training intervention was designed so that for our study sites, the first 10 months were front loaded with training modules relevant to MNH. The intervention used on-site monthly staff training, addressed team-based behaviour, incorporated quality assurance activities and focused on both medical (e.g. evidence-based clinical care) and management (e.g. record keeping, essential medicine monitoring and management) knowledge and practice tools to support implementation of high-quality care. The MNH modules were evidence-based best practices that had been validated and endorsed by the international medical community to address the leading causes of mortality and morbidity for mothers and newborns in Uganda (American Academy of Pediatrics (AAP), 2016; American Congress of Obstetricians and Gynecologists (ACOG), 2015; WHO, 2011; WHO, 2012a,b; WHO, 2015b,c; WHO, 2017a). The trainings were delivered at each facility on a rotating monthly basis. The primary clinical trainer held a bachelor’s degree in public health and a diploma in nursing, while the primary management trainer had a bachelor’s degree in accounting. Trainers delivered these in-facility ∼2 hour long didactic and hands-on modules to the majority of the clinic staff at a designated time and then followed-up with an additional training for any clinicians who could not attend due to running normal operations during the training. Training materials given to staff included partographs and clinical best practice sheets that summarized the training content. The trainings were a mix of lectures, videos, demonstrations and practice (e.g. assembling a condom tamponade to control postpartum haemorrhage (PPH), neonatal resuscitation practice on a dummy, etc.). Each element of LifeNet’s MNH Package aligned with the Ugandan government’s 2016–2020 RMNCAH Sharpened Plan, which emphasizes evidence-based, high-impact health solutions (Republic of Uganda, 2016).

There were 13 MNH-related training modules required by the clinical staff, and non-clinical administrative staff were required to attend at least sessions 1, 3 and 4 as noted below. Modules included (1) documentation and record keeping, (2) partograph and delivery records, (3) basic patient assessment, (4) infection prevention, (5) intravenous (IV) usage, (6) antenatal care, (7) hypertension and pre-eclampsia, (8) first trimester high-risk pregnancies, (9) second and third trimester high-risk pregnancies, (10) normal (uncomplicated) deliveries, (11) PPH, (12) first 5 minutes, APGAR (rapid scoring system for assessing clinical status of newborn), neonatal assessment and (13) neonatal resuscitation. There was significant variability between individual providers and between facilities on session attendance per module ranging from 56 to 100% attendance at individual sessions among relevant staff who should have attended. If new staff joined the clinics mid-evaluation, LifeNet tried to on-board and briefly ‘catch-up’ these new staff on missed trainings. There were no other known QoC interventions happening at the clinics during our study period to the best of our knowledge.

### Study sites

Six rural, faith-based health facilities in the greater Masaka area, new to partnering with LifeNet International, participated in the study. Study facilities were selected based on their proximity to Masaka town (for research supervision purposes) and sufficient obstetric delivery volume (16+ deliveries per month). All facility managers were accredited by the Roman Catholic Diocese of Masaka. Maternal delivery fees averaged ∼30 000 UGX ($8.50 USD). The number of clinical employees per facility ranged from three to nine for all services and between two to six health providers for obstetric services. Clinical providers observed during the study included midwives, comprehensive nurses, clinical officers and doctors. All facilities were designated as able to serve pregnant women with uncomplicated deliveries, including women living with human immunodeficiency virus (HIV), per government guidelines that indicated they must be able to deliver basic emergency obstetric care. One facility was a level IV referral facility capable of performing surgeries including C-sections, with the remaining five facilities being of level III meaning they had no surgical capacity and thus no C-section services (WHO, 2017b). Additional details about study site selection and data collection procedures, including online access to data collection forms, have been published elsewhere (Egger *et al.*, 2020).

### Data collection instruments and procedures

During both the pre- and post-intervention study periods, QoC data were collected using three methods

- Direct Clinical Observation (DCO) of childbirth deliveries documented on a checklist of the clinical encounter.
- Medical record extraction to document the recorded QoC practices and health outcomes (source of data for neonatal and maternal pre-discharge mortality) [‘medical records’ were most often non-standardized ledgers at baseline with very little information routinely captured and then LifeNet-developed medical chart forms at endline], including information from a supplemental 28-day follow-up phone call to consented participants to capture post-discharge neonatal and maternal mortality.
- Facility checklists that were used as a daily audit to track necessary supplies and medicines at each health facility.

Research assistants (RAs), all fluent in English and Luganda, were trained by Duke University, which led the external evaluation, to collect data via direct observation of clinical encounters, review of medical records and daily facility checklists. Ten RAs were deployed for baseline (pre-intervention) data collection and 10 RAs conducted endline (post-intervention) data collection. About half were hired for both timepoints and the RAs reflected a mix of clinical (licensed nurses) and non-clinical health research backgrounds. Study training for the RAs for each timepoint included a 5-day review of LifeNet’s quality improvement training programme delivered by LifeNet staff, followed by a 5-day training in study procedures and research ethics by the university-based study investigators. One to three RAs were assigned per study facility based on delivery volume. RAs were ‘on call’ to respond to all deliveries for observation at their clinics. A shift-schedule ensured that nearly all consecutive maternal deliveries were observed during the study periods. Deliveries not observed were assumed to be missed completely at random.

The DCO form was informed by USAID’s (United States Agency for International Development) Maternal and Child Health Integrated Program (MCHIP) Maternal and Newborn QoC Survey for Labor and Delivery (2013). Study RAs assessed the extent to which providers adhered to best practice standards of care for all stages of maternal deliveries: initial client assessment, first stage of labour, second and third stages of labour, immediate newborn and postpartum care and medical information documentation, including emergency procedures of newborn resuscitation and PPH management.

Data were recorded on paper-based DCO forms and then entered electronically into a Research Electronic Data Capture (REDCap) form. More than one RA could observe a single delivery if RAs changed shifts during a delivery. As such, the RA noted the sections of the delivery process that she observed. Several of the RAs had at least as much clinical training as the providers they observed. To maintain objectivity, the RAs were trained to intervene in the delivery only if they felt that they were critically needed, and if they believed either the life of the mother or the child was in danger. If the RA intervened in any way, this was recorded in the relevant notes section of the DCO form and the respective data were excluded from primary analyses.

Data from medical records were largely from the post-intervention timepoint only and were typically extracted and directly entered into REDCap after the DCO data were entered. Part of the intervention was that clinics were trained to use a more comprehensive two-page medical record form developed by LifeNet in order to document clinical care and health outcomes in a standardized way across sites. While most clinics had access to individual partograph sheets from the Ministry of Health, the partograph was embedded in the LifeNet medical record form so patients had one in their record. During the discharge process, women were asked to provide a contact phone number to be included in their medical chart for the purposes of a brief 28-day follow-up check-in by the RAs or clinic staff.

The facility checklist was also completed daily for each facility. The checklist was informed by the USAID’s MCHIP Facility Inventory Quality of Care Tool (2013a). Study RAs documented availability of resources, support systems and facility infrastructure elements necessary to provide a level of service intended to meet national or international standards. The checklist was completed on paper first and then entered into REDCap.

### QoC indicators

Priority indicators of clinical quality for all deliveries were determined based on the global literature as noted above in the modules and were identified prior to data collection to align with each stage of maternal delivery: initial client assessment, first, second and third stages of labour, immediate newborn and postpartum care and medical information documentation. We also collected observational data on newborn resuscitation and PPH management as the circumstances arose. Adherence to each QoC indicator was defined as the proportion of eligible deliveries where the provider adhered to the recommended best practice and each was measured as a binary response (0/1). For each indicator, the numerator represented the number of deliveries where the provider adhered to best practices and the denominator represented all eligible deliveries. As a result, the denominator (N) differed for each indicator. Our study indicators reflect both ‘sensitive’ indicators, those that require direct observation for confirmation and often have a critical time element to reflect optimal best practice (e.g. maintaining glove sterility and timing of cord clamping) and non-sensitive or ‘crude,’ indicators that could, in theory, have been measured using standard techniques like medical chart abstraction or provider interviews. We believe that sensitive measures may be a better true measure of clinical quality and thus better illuminate potential effects on health impact, than crude measures. Therefore, we created multiple indicators per domain to highlight varying levels of sensitivity.

There are a total of 17 QoC indicators relevant to all women presenting for delivery. For handwashing we have three indicators: provider washes hands at least once during labour and delivery, provider washes hands only once but it is for clean up after birth and provider washes hands at three important timepoints—right before initial vaginal examination, again during the first stage labour and then in preparation for delivery. For sterile glove use, we documented three indicators: use of any type of gloves, use of surgical gloves specifically and then use of surgical gloves without compromising their sterility prior to use. Sterile cord cutting and clamping had three indicators: use of a sterile cord clamp or sterile string to tie off the umbilical cord, use of a sterile blade or sterile scissors to cut the cord, and delayed cord clamping recorded as more than one minute after delivery. For partograph use, we have two indicators: any partograph use, either observed or documented at any time and partograph used in real-time to monitor labour. To prevent PPH, administration of a uterotonic during the second and third stages of labour was documented, and as part of best practice during initial assessment, testing the woman’s urine for the presence of protein was observed. Appropriate use and timing of the APGAR is best practice, and we had documented use at one and/or 5 minutes post-delivery via medical record documentation and/or observation.

### Sample size and power calculations

Our study was powered on the primary aim of estimating a difference in the prevalence of the QoC indicator, ‘real-time proper use of the partograph’, comparing the baseline and endline periods. With an expected 155 maternal deliveries observed in each of the two time periods, we estimated achieving greater than 80% power to detect a difference as small as 14% in the proportion of those encounters where the partograph was used properly when the baseline (i.e. ‘pre-’) proportion is estimated to be 20% (20 vs 34%, based on a Chi-squared test of independence, assuming a two-sided alpha level of 0.05). Our power was estimated to reach 90% if the difference in the two proportions was as large as or larger than 16.5%. We expected to see similar, if not higher, power on most of our other indicators. If the baseline proportion of the partograph indicator was lower than 20%, then our estimates of power were conservative. The study exceeded our target sample size requirements and achieved a priori statistical power to meet all of our primary research aims.

### Data management and analysis

Data were analysed using Stata 16 software (StataCorp, College Station, TX). Analysis of study data focused on the change in the prevalence (i.e. the adherence) from baseline (pre-intervention) to endline (post-intervention). Data were analysed separately at the individual (i.e. patient-provider encounter) and clinic cluster levels and results were compared. Individual-level analysis utilized a generalized estimating equation (GEE) framework to account for clustering in the response by clinic and assumed an exchangeable working correlation structure. Models assuming an independent working correlation structure were fit as a sensitivity analysis. The GEE model was fit to prevalent data with a log-link function using a modified Poisson regression approach (Zou, 2004). Models were fit in Stata using the xtgeebcv command, which allows for a finite sample bias correction due to the small number of clusters in our study (Gallis *et al.*, 2020). We used the bias-corrected variance method proposed by Kauermann and Carroll (2001). Models included an indicator parameter for time (i.e. baseline vs endline), and exponentiation of this parameter estimated the population averaged prevalence ratio (PR). A positive value for the PR can be interpreted as an average increase in adherence over the study period. A negative value for the PR can be interpreted as an average decrease in the adherence over the study period. Cluster-level analyses were performed in MS Excel using methods for pair-matched clusters described in Hayes and Moulton (2017).

Ethical approvals were obtained from [Duke University U.S. Academic Institution], The AIDS Support Organization in Uganda [not affiliated with authors], and the Uganda National Council of Science and Technology. All maternity clients (or her self-designated proxy) gave written informed consent to participate in the study and were provided with a copy of the consent form with contact information. Consent was obtained at admission for childbirth; however, our study team also confirmed consent again post-delivery. No participant rescinded their consent.

## Results

To appropriately assess clinical quality practices, facility infrastructure and resources were assessed daily at each study clinic during the pre- and post-direct observation periods (Table 1). There were 364 observed facility days (6 clinics, ∼60 days each) at baseline and 424 observed facility days (6 clinics, ∼70 days each) at endline. Overall, facility infrastructure was relatively high with little variation in the availability of equipment and supplies, although there were some notable improvements from baseline to endline. There was one clinic in particular that drove some of the lower levels of available supplies and equipment at baseline, moving from 0 to 100% on a few indicators (e.g. oral thermometer and IV equipment). Other notable changes include the percentage of clinic days with sterile cloths doubling over time (17 to 31%) but still low, and the availability of condom tamponade packages during a third of observed facility days at endline (35%), compared to almost none at baseline.

**Table 1.** Daily Facility Infrastructure Assessment, pre- and post-intervention at six study health facilities

	Baseline (*n* = 364 observed facility days)	Endline (*n* = 424 observed facility days)
Health system quality characteristics	%	%
Skilled birth attendant accessible	98.6	99.3
Any power loss in past 24 hours	23.1	14.2
General equipment and supplies		
Maternal stethoscope	49.2	84.7
Newborn resuscitation mask	99.7	100
Rectal thermometer for newborn	0	0.7
IV materials (catheter for IV line, infusion stand and IV cannula)	100	99.8
Urinary catheters	83.7	88.4
Oral thermometer	83.2	99.5
Adult ventilator bag and mask	67.0	54.0
PPH items		
Sterile cloth	17.0	30.6
Sterile gloves	100	83.2
Condom tamponade packages (at least 2)	0.3	34.7
IV giving set	83.2	83.7
Ligature, pack of sutures	83.2	77.8
Bag of 500 ml IV fluids (at least 2)	66.2	83.7
Long clamp, 25–50 cm long	82.7	83.3
Tests		
Glucometer	91.2	83.7
Urinalysis test strips	100	99.8
Determine HIV rapid testing kit	87.6	100
Syphilis test	99.7	83.7
Pharmacy		
Functional and adequate refrigeration	93.7	98.6
Expired drugs, any	15.4	2.1
Nevirapine syrup	80.1	98.1
Vitamin K	100	83.0
Vitamin A	82.6	100
Oxytocin or misoprostol	100	100
Antibiotic, any	100	100
Anticonvulsant, any	100	100
Hypertensive, any	100	100
Antiretrovirals (ARVs), any	87.9	99.8
IV fluids	99.4	100

For the baseline (pre-intervention) assessment, 263 deliveries were consented and observed (representing 94% of all women presenting for delivery) and 228 women (87%) were followed up 28 days later for questions on maternal and neonatal mortality. For the endline (post-intervention) follow-up assessment, 321 deliveries were consented and observed (representing 90% of all women presenting for delivery) and 304 women (95%) were followed up 28 days later for mortality data.

Results of individual level analysis, estimating the PR for each of the 17 clinical quality indicators and comparing the adherence pre- and post-intervention are presented in Table 2. For all 15 of the indicators where we were able to estimate a model-based PR, we observed a PR of greater than 1.0, indicating a positive trend toward improved adherence. We observed a significant increase in the prevalence of adherence over the study period for three indicators (Table 2). These increases were large and clinically meaningful; however, there was considerable variation in adherence by clinic for many indicators, as indicated by the wide confidence intervals. Overall, for the indicators ‘handwashing at least once during initial assessment, labour and/or in preparation for delivery’, ‘delayed cord clamping’ and ‘any partograph documentation,’ we observed increases indicating that the average provider was 4.79, 2.48 and 7.99 times more likely to perform these three procedures post-implementation of the LifeNet training, respectively.

**Table 2.** Results of fitting univariable GEE log binary regression to estimate the effect of the LifeNet's clinical training programme^a^ in the greater Masaka area of Uganda [PR]

QoC indicator	Baseline *n*	Endline *n*	PR^b^	95% CI
Hand washing				
Provider washed hands at least once right before initial vaginal exam and/or during first stage of labor and/or preparation for delivery	177	205	**4.79**	**1.02, 22.53**
Provider washed hands only once for clean-up only after birth	217	259	1.27	0.76, 2.15
Provider washed hands all three times—right before initial vaginal exam, during first stage of labor and preparation for delivery	177	205	—^d^	—
Sterile glove use				
Use of any gloves during the second and third stage of labor for vaginal examination/delivery	228	265	—^d^	—
Pre-packaged, surgical gloves used for vaginal examination/delivery	223	262	1.05	0.69, 1.61
Provider did not compromise the sterility of gloves	209	257	1.53	0.51, 4.62
Sterile cord cutting and clamping				
Use of sterile cord clamp or sterile string during immediate newborn and postpartum care	226	259	1.19	0.82, 1.75
Use of sterile blade or scissors to cut cord during immediate newborn and postpartum care	221	259	1.15	0.73, 1.83
Delayed cord clamping (>1 minute)	196	246	2.48	1.31, 4.72
Partograph use				
Any partograph documentation, either observed or documented	222	264	4.01	1.57, 10.25
Partograph used in real-time to monitor labor	221	264	4.73	0.36, 61.92
Uterotonic use for prevention of PPH				
Use of uterotonic during second and third stage of labor	228	262	1.01	0.86, 1.19
Urine testing				
Mother’s urine tested for presence of protein during initial client assessment	235	291	1.73	0.49, 6.07
APGAR				
Documented APGAR score at 1, OR 5 minutes during chart review	263	296	1.20	0.83, 1.74
Documented APGAR score at 1, AND 5 minutes during chart review	263	295	1.30	0.51, 3.32
Provider was observed to conduct APGAR score at 1 OR 5 minutes	204	259	7.99	0.08, 816.80^c^
Provider was observed to conduct APGAR score at 1 AND 5 minutes	204	259	23.12	0.22, 2411.24^c^

a Training modules that covered key QoC indicators (although messages could be re-emphasized in subsequent modules): handwashing and sterile glove—modules 4, 10 and 11; sterile cord cutting and clamping—module 12; partograph use—modules 2 and 10; uterotonic use for prevention of PPH—modules 10 and 11; urine testing—module 7; APGAR—Modules 10 and 12.

b GEE-based log-risk models were fit to data using a variance correction to account for the small number of clinics in the data. Due to convergence issues, all models are unadjusted.

c Due to zero cell totals indicating complete lack of adherence, estimated variance is very high and these results should be interpreted with caution.

d Inestimable due to model non-convergence.

Adherence data for each of the 17 clinical quality indicators, stratified by clinic and time period, is presented in Table 3. There was considerable variation in adherence across clinics for each of the indicators. Due to zero cell counts (e.g. the provider adhered to the indicator 0 times in a clinic at a specific time point), we were only able to estimate the cluster-level PR for 6 of the 17 indicators. However, results for these six indicators are consistent with results from the individual-level analysis, showing a trend of improved adherence comparing baseline to endline.

**Table 3.** Adherence to clinical quality indicators at baseline (BL) and endline (EL), by clinic, with cluster level PR where estimable, in the greater Masaka area in Uganda

		Baseline		Endline						
QoC indicator	Clinic	Adhered (#)	Total (#)	Adhered (#)	Total (#)	Prev_BL_	Prev_EL_	PR		
Hand washing										
Provider washed hands at least once right before initial vaginal exam and/or during first stage of labour and/or preparation for delivery										
	1	2	13	1	25	0.15	0.04			
	2	0	19	4	19	0.00	0.21			
	3	4	39	48	54	0.10	0.89			
	4	3	19	22	24	0.16	0.92			
	5	1	28	0	36	0.04	0.00			
	6	2	59	1	47	0.03	0.02			
Provider washed hands only once for clean-up only after birth										
	1	23	23	8	26	1.00	0.31	0.31		
	2	4	23	13	19	0.17	0.68	3.93		
	3	48	50	63	63	0.96	1.00	1.04		
	4	6	26	34	34	0.23	1.00	4.33	Pooled PR^a^	1.40
	5	19	30	26	39	0.63	0.67	1.05	PR (95%-)	0.50
	6	47	65	75	78	0.72	0.96	1.33	PR (95%+)	3.93
Provider washed hands all three times: right before initial vaginal exam, during first stage of labour and preparation for delivery										
	1	0	13	0	25	0.00	0.00			
	2	0	19	1	19	0.00	0.05			
	3	0	39	34	54	0.00	0.63			
	4	0	19	12	24	0.00	0.50			
	5	0	28	0	36	0.00	0.00			
	6	0	59	0	47	0.00	0.00			
Sterile glove use										
Use of any gloves during the second and third stages of labour for vaginal examination/delivery										
	1	23	23	27	27	1.00	1.00	1.00		
	2	24	24	20	20	1.00	1.00	1.00		
	3	52	52	66	66	1.00	1.00	1.00		
	4	28	28	34	34	1.00	1.00	1.00	Pooled PR	1.00
	5	36	36	39	39	1.00	1.00	1.00	PR (95%-)	0.99
	6	65	65	78	79	1.00	0.99	0.99	PR (95%+)	1.00
Pre-packaged, surgical gloves used for vaginal examination/delivery										
	1	23	23	27	27	1.00	1.00	1.00		
	2	21	24	20	20	0.88	1.00	1.14		
	3	49	52	66	66	0.94	1.00	1.06		
	4	24	27	33	34	0.89	0.97	1.09	Pooled PR	1.06
	5	31	32	39	39	0.97	1.00	1.03	PR (95%-)	1.00
	6	61	65	73	76	0.94	0.96	1.02	PR (95%+)	1.11
Provider did not compromise the sterility of gloves										
	1	1	23	13	26	0.04	0.50			
	2	2	21	10	20	0.10	0.50			
	3	11	49	29	66	0.22	0.44			
	4	0	24	1	33	0.00	0.03			
	5	13	31	13	39	0.42	0.33			
	6	10	61	4	73	0.16	0.05			
Sterile cord cutting and clamping										
Use of sterile cord clamp OR sterile string during immediate newborn and postpartum care										
	1	23	23	26	26	1.00	1.00			
	2	0	23	14	19	0.00	0.74			
	3	49	51	61	63	0.96	0.97			
	4	4	28	0	34	0.14	0.00			
	5	30	36	33	39	0.83	0.85			
	6	51	65	78	78	0.78	1.00			
Use of sterile blade or scissors to cut cord during immediate newborn and postpartum care										
	1	23	23	26	26	1.00	1.00	1.00		
	2	23	23	19	19	1.00	1.00	1.00		
	3	50	50	63	63	1.00	1.00	1.00		
	4	20	25	33	34	0.80	0.97	1.21	Pooled PR	1.13
	5	32	35	38	39	0.91	0.97	1.07	PR (95%-)	0.92
	6	39	65	77	78	0.60	0.99	1.65	PR (95%+)	1.39
Delayed cord clamping (>1 minute)										
	1	2	16	4	24	0.13	0.17	1.33		
	2	11	19	18	19	0.58	0.95	1.64		
	3	10	44	50	57	0.23	0.88	3.86		
	4	11	27	29	34	0.41	0.85	2.09	Pooled PR	2.09
	5	11	30	22	39	0.37	0.56	1.54	PR (95%-)	1.35
	6	16	60	59	73	0.27	0.81	3.03	PR (95%+)	3.23
Partograph use										
Any partograph documentation, either observed or documented										
	1	1	23	17	33	0.04	0.52			
	2	0	21	10	21	0.00	0.48			
	3	24	52	67	76	0.46	0.88			
	4	0	23	22	27	0.00	0.81			
	5	5	38	17	43	0.13	0.40			
	6	18	65	60	64	0.28	0.94			
Partograph used in real time to monitor labour										
	1	0	22	4	33	0.00	0.12			
	2	0	21	10	21	0.00	0.48			
	3	20	52	39	76	0.38	0.51			
	4	0	23	3	27	0.00	0.11			
	5	2	38	2	43	0.05	0.05			
	6	1	65	48	64	0.02	0.75			
Uterotonic use for prevention of PPH										
Use of uterotonic during second and third stage of labour										
	1	22	23	24	26	0.96	0.92	0.97		
	2	19	24	15	20	0.79	0.75	0.95		
	3	52	52	61	65	1.00	0.94	0.94		
	4	28	28	34	34	1.00	1.00	1.00	Pooled PR	1.00
	5	34	36	39	39	0.94	1.00	1.06	PR (95%-)	0.94
	6	60	65	78	78	0.92	1.00	1.08	PR (95%+)	1.06
Urine testing										
Mother’s urine tested for presence of protein during initial client assessment										
	1	0	14	5	36	0.00	0.14			
	2	0	26	0	23	0.00	0.00			
	3	0	70	2	91	0.00	0.02			
	4	0	26	0	35	0.00	0.00			
	5	0	33	2	39	0.00	0.05			
	6	5	66	2	67	0.08	0.03			
APGAR										
Documented APGAR score at 1, OR 5 minutes during documentation review										
	1	23	26	25	28	0.88	0.89			
	2	0	27	19	20	0.00	0.95			
	3	64	70	86	91	0.91	0.95			
	4	27	31	33	35	0.87	0.94			
	5	32	39	41	43	0.82	0.95			
	6	65	70	78	79	0.93	0.99			
Documented APGAR score at 1 AND 5 minute during documentation review										
	1	22	26	20	29	0.85	0.69			
	2	0	27	18	20	0.00	0.90			
	3	64	70	82	89	0.91	0.92			
	4	0	31	24	35	0.00	0.69			
	5	5	39	27	43	0.13	0.63			
	6	63	70	52	79	0.90	0.66			
Provider was observed to conduct APGAR score at 1 OR 5 minutes										
	1	1	1	2	26	1.00	0.08			
	2	0	23	19	19	0.00	1.00			
	3	0	51	37	63	0.00	0.59			
	4	1	28	30	34	0.04	0.88			
	5	20	36	1	39	0.56	0.03			
	6	0	65	78	78	0.00	1.00			
Provider was observed to conduct APGAR score at 1 AND 5 minutes										
	1	1	1	0	26	1.00	0.00			
	2	0	23	19	19	0.00	1.00			
	3	0	51	18	63	0.00	0.29			
	4	0	28	1	34	0.00	0.03			
	5	3	36	0	39	0.08	0.00			
	6	0	65	47	78	0.00	0.60			

a The cluster-level pooled PR is calculated using the method described in Hayes and Moulton (Hayes & Moulton, 2017) assuming a matched pair analysis.

Due to the expected rarity of mortality, the study was not powered to detect significant differences in neonatal and maternal mortality; nonetheless, we report the deaths that were observed as valuable descriptive data including information on stillbirths, health status at discharge and health status up to 28 days postpartum (Table 4). The data indicate a decreased pre-discharge neonatal mortality prevalence at the conclusion of the LifeNet training intervention. During the pre-intervention study period, there were seven pre-discharge neonatal deaths out of 263 observed live births that indicates a pre-discharge neonatal mortality prevalence of 0.0268 (95% CI (exact): 0.011, 0.054) at baseline. During the post-intervention study period, there were three pre-discharge neonatal deaths out of 321 observed live births, which indicates a pre-discharge neonatal mortality prevalence of 0.009 (95% CI (exact): 0.002–0.027) at endline. The 28-day neonatal mortality at baseline was 0.042 (95% CI (exact): 0.021, 0.074) (*n* = 11) and at endline it was 0.016 (95% CI (exact): 0.005, 0.036) (*n* = 5).

**Table 4.** Obstetric deliveries and MNH outcomes

	Baseline	Endline
Total number of deliveries	*n* = 263	*n* = 321
Number of observed deliveries per study clinic (range)	26–70	24–92
Mean age of mother (standard deviation)	24.7 (5.5)	24.8 (6.1)
Nighttime deliveries (18:00 to 06:00)	120 (45.6%)	160 (49.8%)
Late night deliveries (01:00 to 5:59)	45 (17.1%)	70 (21.8%)
Maternal morbidity and mortality	*n* = 257^a^	*n* = 318^a^
PPH	8 (3.1%)	5 (1.6%)
Discharged in poor health, emergency referral (mother)	27 (10.5%)	23 (7.2%)
Maternal deaths (prior to discharge)	0	0
Maternal deaths (up to 28 days)	0	1^b^
Neonatal morbidity and mortality	*n* = 254^a^	*n* = 314^a^
Fresh stillbirths	2 (0.8%)	0
Macerated stillbirths	0	2 (0.6%)
Discharged in poor health, emergency referral (child)	21 (8.3%)	9 (2.9%)
Early neonatal deaths (prior to discharge)	7 (2.8%)	3 (1.0%)
Neonatal deaths (up to 28 days)	11 (4.3%)	5 (1.6%)

a The *n* subtotals are exclusive of missing data.

b Mother discharged with emergency referral, died at referring hospital, twin birth.

During the baseline period, there were zero pre-discharge maternal deaths out of 263 observed deliveries. During the endline period, there was one maternal death out of 321 observed deliveries but it was not documented pre-discharge. The labouring woman was discharged from the LifeNet partner clinic in poor condition and died upon arrival at the next referral hospital to which she was transferred (it was a complicated twin delivery).

During the baseline period, 19 of 24 (79%) cases of attempted neonatal resuscitation were successful. During the endline period, 13 of 14 (93%) attempted resuscitations were successful. The vast majority of cases at both baseline and endline were observed to conduct appropriate resuscitation techniques, including suctioning the airway, rubbing the back, positioning the head, ventilating, etc.

Data collectors observed and documented QoC for managing PPH if it occurred. At baseline, there were eight recorded observations of PPH. In all eight instances, bleeding was monitored, uterine massage was performed, uterotonic was given and the provider performed an abdominal exam for uterine contraction and examined the vagina and perineum for lacerations or cervical tear. Three providers examined the placenta for completeness, six started IV fluids, three performed uterine exploration, none used mechanical evacuation or manual removal of the placenta, two performed aortic compression, none used balloon or condom tamponade, two used uterine sutures, five gave antibiotics and seven raised the woman’s legs at some point after the PPH began. PPH causes listed in the medical records included coagulopathy, laceration, atonic uterus, incomplete expulsion of placenta and retained placenta.

At endline, there were five recorded observations of PPH. In all five instances, bleeding was monitored, uterine massage was performed, uterotonic was given and the provider performed an abdominal exam for uterine contraction and examined the vagina and perineum for lacerations or cervical tear. Three providers examined the placenta for completeness, two started IV fluids, none performed uterine exploration, two used mechanical evacuation or manual removal of the placenta, one performed aortic compression, none used balloon or condom tamponade, none used uterine sutures, one gave antibiotics and one raised the woman’s legs at some point after the PPH began. PPH causes listed in the medical records included incomplete expulsion of placenta, atonic uterus and retained placenta.

## Discussion

The global calls for improved QoC for MNH align with recognition that service coverage is not enough for an impact on health outcomes (Countdown to 2030 Collaboration, 2018; WHO, OECD & International Bank for Reconstruction and Development. 2018). High-quality intrapartum facility-based care is critical for reducing maternal and early neonatal mortality, and accountability and action are guiding purposes of quality measurement (Kruk *et al.* 2018). This evaluation indicates that the LifeNet training intervention significantly improved maternal and neonatal healthcare quality on several key indicators and supported general positive trends across all indicators, at all six primary care clinics in Uganda, at least over a relatively short-term period. The QoC indicators reflect gold standard measurement via direct observation and are robust; however, for several of these quality indicators, the adherence rates were far from 100% and could still benefit from further improvement.

Clinical social franchising is a rapidly expanding intervention in the private sector for improving QoC and increasing utilization of quality services via branded, quality-assured services, yet their impact has been mixed (Montagu *et al.*, 2016; Beyeler *et al.*, 2013). However, external evaluations of these organizations, such as LifeNet International, are critical for objectively documenting impact given the pressure of sustainability (both for the franchisor and franchisee perspective) these private sector efforts face. There are many clinical QoC training initiatives but fewer models that focus on sustained engagement with partner clinics with evidence of impact over time (Rowe *et al.*, 2018).

The study results, while largely positive and clinically meaningful, highlight tremendous shortcomings in QoC. LifeNet International’s earliest training modules focus on infection control and handwashing as a core best practice. Despite the lessons and reviews, only two of the six clinics were observed to have providers who washed their hands at least once before and/or during deliveries the majority of the time at endline. Qualitative insights during the study on this suboptimal practice included provider confidence in and reliance upon gloves (without understanding how glove sterility could be comprised) and difficulties drying hands prior to putting on gloves. These nuanced issues could be addressed via a refresher training; however, if structural issues, such as lack of towels for drying hands are not addressed, training alone will not succeed. Furthermore, if the evaluation had relied on provider self-report for handwashing, adherence estimates would have been seriously biased as most providers did wash their hands, but only after delivery for clean-up purposes, which has no effect on the sterility of the delivery.

We observed a large and clinically meaningful increase in real-time partograph use over the study period; however, this government-endorsed best practice is still underutilized. The evidence of partograph efficacy in reducing maternal and neonatal deaths is mixed (Lavender *et al.*, 2018); but if the government of Uganda and the WHO continue to recommend its use and LifeNet trains on it, further work should be done to understand why providers fail to utilize it during deliveries. A review of barriers to real-time use of the partograph in low-resource settings revealed many reasons including lack of graphing skills, stockouts, an organizational acceptance of retrospective documentation, and providers not internalizing its function and value (Ollerhead and Osri, 2014). We expect the training by LifeNet could have impacted multiple barriers, and future monitoring by LifeNet could include open-ended questions to providers to better understand uptake or lack thereof. Additional qualitative data more broadly could have helped us better understand why some indicators had more dramatic improvements than others.

The issue of medical documentation during and not after providing clinical services is an important one. Most of our study clinics only had one or two clinical staff available to provide maternity services and time was precious. In addition, as noted earlier, half of the deliveries occurred at night and power losses were common. Real-time, appropriate medical documentation was likely impeded by both physical constraints and provider prioritization issues. We received informal qualitative feedback from our local study team that there were strong indications that the LifeNet medical record itself improved clinical practice. Prior to our study, clinics typically wrote minimal information about each delivery in a ledger. For the purposes of the study, we needed to standardize the medical records, and in the process, we enhanced the type and quality of information required for documentation. For example, the record asked what was used to cut the umbilical cord, how many minutes after birth was the cord clamped and whether breastfeeding was initiated within 1 hour, all of which would not have been typically recorded in a ledger. That said, we have evidence that the medical records were often not valid measures of clinical quality as they often did not reflect directly observed practice (Kim *et al.*, 2021), tending to overestimate good clinical practice. Even with these limitations, we recommend utilization of these more detailed, checklist-type medical records as they appear to also serve as prompts for high-quality practices (Rowe *et al.*, 2018; Tolu *et al.*, 2020). Because our study collected data using this medical chart during both the baseline and endline periods, our measured effect of the LifeNet intervention (i.e. PRs) is very likely to be conservative since it does not account for the effect of the medical chart itself in improving clinical quality.

Our effect estimates should also be interpreted more as measures of effectiveness than efficacy since we did not attempt to control compliance of providers to the intervention. Staff turnover at the clinics, particularly at the provider and in-charge levels, may have minimized or negated the effect of LifeNet’s clinical training on some indicators. Between baseline and endline, our study team was tracking implementation of the training modules against staff attendance and realized that LifeNet did not have a routinely monitored on-boarding process for catching up staff on past training lessons. Near the end of the 10 month intervention period, LifeNet course-corrected by strengthening their monitoring system to properly track whether newly hired staff were individually caught up on missed content. Likewise, poor attendance to LifeNet’s training by key clinic staff, such as the in-charge, may have minimized the buy-in to the trainings and the training’s overall effects. Potential under-compliance with the LifeNet trainings by medical providers likely led to effect estimates that are biased conservatively towards the null.

### Study limitations

This study had several limitations. First, because we were unable to include a randomly assigned control group over the same time period as the intervention (e.g. an randomized controlled trial [RCT]), we cannot infer causality with respect to the intervention on improving QoC. For example, while the LifeNet staff and our study team monitored for other maternal and neonatal interventions during the study period and did not identify any, there is still a chance something could have been implemented in the study clinics at the same time as our study period that affected healthcare quality. Furthermore, while most providers were assessed at both baseline and endline, some new providers were included in endline who were not included in baseline. Any differences in provider-level characteristics (e.g. time of relevant experience) that may have varied across study periods and which are prognostic of the outcome (QoC measures) may have confounded the effect of the intervention. We attempted to control for the potential confounding effect of study clinic (i.e. the distribution of clinical encounters varied by both clinic and time period); however, due to many zero cell sizes, our adjusted models did not converge, and so we present unadjusted effect estimates only. However, we separately present results of cluster-level analyses that match on clinic, which eliminates any confounding of the PR by clinic. Since these cluster-level PR estimates were very similar to our individual level estimates we do not believe confounding by clinic is of significant concern to the validity of our estimates.

Although these factors affect our causal inference, the timing of changes in outcomes and the positive trends and significance of the changes across many indicators specific to LifeNet’s training, considered alongside the monitoring of information on the (lack of) other interventions that may have occurred at study clinics, lead us to believe the general magnitude and direction of our estimates, and any measurable improvements in adherence to these indicators are likely attributable to the LifeNet training.

Second, our team had to review and select priority indicators without accepted global guidance on what core indicators best represent QoC. There are now efforts underway to validate and standardize selected observational measures of quality for newborn and maternal facility-based care, which may better reflect indicators that influence health impact (Day *et al.*, 2019). In addition, we did not capture an important domain of QoC, respectful maternity care (RMC) that is now clearly part of new WHO guidelines on a positive childbirth experience (WHO, 2018). LifeNet did not have an RMC-specific module at the time of the study (they did address what they called patient-centred, compassionate care), and thus, we did not measure it. As an increasingly important aspect of high QoC that could have implications for other MNH indicators, we highly recommend it be included in future trainings based on new guidance coming from WHO and associated monitoring indicators. One hint about the clinical environment that we noticed is that patients were much more willing to share their phone numbers with our RAs (with whom they built some rapport during the direct observation of their delivery) than with the providers in order to assess 28-day health outcomes. This could reflect a patient–provider communication gap that could be further strengthened.

And finally, as noted earlier, this study was not powered for mortality outcomes. Even though we documented a reduction in the pre-discharge risk of death, comparing the baseline to the endline periods, our estimates are not precise, and thus, this difference may be real or it may be due to random chance.

### Study strengths

The most notable strength of the study was our use of direct observation. By not relying on medical records or provider self-report, we have reduced the potential bias of over-estimating impact. For example, in a companion article from this study, we estimated the average specificity of our QoC indicators at only 34% when using medical records instead of direct observation (Kim *et al.*, 2021). In addition, the study was designed as a ‘real-world’ impact evaluation, and accordingly, we note that the study clinics experienced some clinical staff turnover that is typical in a health system. Any impact from the training intervention should reflect the impact under a realistic clinical context. The study QoC indicators were also practice-oriented and did not focus on provider knowledge. This was intentional as increases in knowledge often do not translate into sustained changes in practice and therefore would not be impactful for the study clinics (Bang *et al.*, 2016; Rowe *et al.*, 2018).

### Future research or policy implications

Both public and private sector clinical QoC training interventions need a robust evidence base in order to scale and achieve impact in low-resource settings. As the MNH field struggles with the knowledge–practice gap, more research is needed to better understand whether more sensitive measures of clinical quality, captured by intensive data collection procedures, such as direct clinical observation, lead to better health outcomes when programmes have access to higher-quality clinical practice data. This study indicates that a social franchised-based clinical quality training, like the one performed by LifeNet International, provides clinically significant improvements in maternal and neonatal healthcare, at least over the short term. Future evaluations should focus on the durability of these effects over longer time periods, including impacts on morbidity and mortality, taking into full consideration issues related to training compliance, staff turnover and continuing medical education and refresher trainings.

## Data Availability

Data are available from the corresponding author upon reasonable request.

## References

[R1] American Academy of Pediatrics (AAP). 2016.*Helping Babies Breathe, 2nd Edition Update Guide*. https://www.aap.org/en-us/Documents/hbs_2ndedition_updateguide.pdf.

[R2] American Congress of Obstetricians and Gynecologists (ACOG). 2015.*The APGAR Score*. https://www.acog.org/clinical/clinical-guidance/committee-opinion/articles/2015/10/the-apgar-score, accessed 30 August 2020.

[R5] Bang A, Patel A, Bellad R et al. 2016. Helping Babies Breathe (HBB) training: what happens to knowledge and skills over time? *BMC Pregnancy and Childbirth* 16: 364. 10.1186/s12884-016-1141-3 PMC5120476 27875999

[R6] Beyeler N, Cruz AYDL, Montagu D 2013. The impact of clinical social franchising on health services in low- and middle-income Countries: a systematic review. *PLoS One* 8: e60669. 10.1371/journal.pone.0060669 PMC3634059 23637757

[R7] Campbell OMR et al. 2016. The scale, scope, coverage, and capability of childbirth care. *The Lancet* 388: 2193–208. 10.1016/S0140-6736(16)31528-8 27642023

[R8] Conlon CM et al. 2019. Saving mothers, giving life: it takes a system to save a mother (Republication). *Global Health: Science and Practice* 7: 20–40. 10.9745/GHSP-D-19-00092 PMC6538123 30926736

[R9] Countdown to 2030 Collaboration. 2018. Countdown to 2030: tracking progress towards universal coverage for reproductive, maternal, newborn, and child health. *Lancet (London, England)* 391: 1538–48. 10.1016/S0140-6736(18)30104-1 29395268

[R10] Day LT et al. 2019. “Every Newborn-BIRTH” protocol: observational study validating indicators for coverage and quality of maternal and newborn health care in Bangladesh, Nepal and Tanzania. *Journal of Global Health* 9: 010902. 10.7189/jogh.09.01902 PMC6406050 30863542

[R0010a] Egger JR, Headley J, Li Y, et al. 2020. Beneath the Surface: A Comparison of Methods for Assessment of Quality of Care for Maternal and Neonatal Health Care in Rural Uganda. *Maternal and child health journal* 24: 328–339. 31894511 10.1007/s10995-019-02862-w

[R11] Evans CL et al. 2018. Peer-assisted learning after onsite, low-dose, high-frequency training and practice on simulators to prevent and treat postpartum hemorrhage and neonatal asphyxia: a pragmatic trial in 12 districts in Uganda. *PLoS One* 13: e0207909. 10.1371/journal.pone.0207909 PMC6296740 30557350

[R12] Gallis JA, Li F, Turner EL. 2020. xtgeebcv: a command for bias-corrected sandwich variance estimation for GEE analyses of cluster randomized trials. *The Stata Journal* 20: 363–81. 10.1177/1536867x20931001 PMC8942127 35330784

[R13] Republic of Uganda. 2016. Global Financing Facility.*Investment Case for Reproductive, Maternal, Newborn, Child and Adolescent Health Sharpened Plan for Uganda (2016/17 –2019/20)*. https://www.globalfinancingfacility.org/investment-case-reproductive-maternal-newborn-child-and-adolescent-health-sharpened-plan-uganda, accessed 15 November 2019.

[R14] Hayes RJ, Moulton LH. 2017. Chapman and Hall/CRC biostatistics series. *Cluster Randomised Trials*. 2nd edn. Boca Raton, FL: CRC Press.

[R15] Hirose A et al. 2018. Technical quality of delivery care in private- and public-sector health facilities in Enugu and Lagos States, Nigeria. *Health Policy and Planning* 33: 666–74. 29684122 10.1093/heapol/czy032

[R16] Kara N et al. 2017. The betterbirth program: pursuing effective adoption and sustained use of the WHO safe childbirth checklist through coaching-based implementation in Uttar Pradesh, India. *Global Health: Science and Practice* 5: 232–43. 10.9745/GHSP-D-16-00411 PMC5487086 28655801

[R17] Kauermann G, Carroll RJ. 2001. A note on the efficiency of sandwich covariance matrix estimation. *Journal of the American Statistical Association* 96: 1387–96.

[R0017a] Kim MK, Baumgartner JN, Headley J et al. 2021. Medical record bias in documentation of obstetric and neonatal clinical quality of care indicators in Uganda. *Journal of Clinical Epidemiology* 136: 10–19. 33667620 10.1016/j.jclinepi.2021.02.024

[R18] Koblinsky M et al. 2016. Quality maternity care for every woman, everywhere: a call to action. *The Lancet* 388: 2307–20. 10.1016/S0140-6736(16)31333-2 27642018

[R19] Kruk ME et al. 2018. High-quality health systems in the Sustainable Development Goals era: time for a revolution. *The Lancet Global Health* 6: e1196–252.doi: 10.1016/S2214-109X(18)30386-3. 30196093 PMC7734391

[R20] Kruk ME, Vail D, Austin-Evelyn K et al. 2016. Evaluation of a maternal health program in Uganda And Zambia finds mixed results on quality of care and satisfaction. *Health Affairs* 35: 510–9. 26953307 10.1377/hlthaff.2015.0902

[R21] Lavender T, Cuthbert A, Smyth RM 2018. Effect of partograph use on outcomes for women in spontaneous labour at term and their babies. *The Cochrane Database of Systematic Reviews* 2018: 8. 10.1002/14651858.CD005461.pub5 PMC6513424 30080256

[R22] Lawn JE et al. 2014. Every newborn: progress, priorities, and potential beyond survival. *The Lancet* 384: 189–205. 10.1016/S0140-6736(14)60496-7 24853593

[R23] McPake B, Hanson K. 2016. Managing the public–private mix to achieve universal health coverage. *The Lancet* 388: 622–30. 10.1016/S0140-6736(16)00344-5 27358252

[R24] Montagu D et al. 2016. Recent trends in working with the private sector to improve basic healthcare: a review of evidence and interventions. *Health Policy and Planning* 31: 1117–32. 27198979 10.1093/heapol/czw018

[R25] Olivier J et al. 2015. Understanding the roles of faith-based health-care providers in Africa: review of the evidence with a focus on magnitude, reach, cost, and satisfaction. *The Lancet* 386: 1765–75. 10.1016/S0140-6736(15)60251-3 26159398

[R26] Ollerhead E, Osri D. 2014. Barriers to and incentives for achieving partograph use in obstetric practice in low- and middle-income countries: a systematic review. *BMC Pregnancy and Childbirth* 14: 281. 10.1186/1471-2393-14-281 PMC4147181 25132124

[R27] Rowe AK, Rowe SY, Peters DH et al. 2018. Effectiveness of strategies to improve health-care provider practices in low-income and middle-income countries: a systematic review. *The Lancet Global Health* 6: e1163–75.doi: 10.1016/S2214-109X(18)30398-X. 30309799 PMC6185992

[R28] Semrau KE et al. 2017. Outcomes of a coaching-based who safe childbirth checklist program in India. *The New England Journal of Medicine* 377: 2313–24. 29236628 10.1056/NEJMoa1701075 PMC5672590

[R29] Tolu LB, Jeldu WG, Feyissa GT. 2020. Effectiveness of utilizing the WHO safe childbirth checklist on improving essential childbirth practices and maternal and perinatal outcome: A systematic review and meta-analysis. *PLoS One* 15: e0234320.doi: 10.1371/journal.pone.0234320. PMC7292415 32530940

[R30] Tunçalp Ö et al. 2015. Quality of care for pregnant women and newborns—the WHO vision. *BJOG: An International Journal of Obstetrics and Gynaecology* 122: 1045–9. 25929823 10.1111/1471-0528.13451 PMC5029576

[R31] Uganda Bureau of Statistics (UBOS) and ICF. 2018. *Uganda Demographic and Health Survey 2016*. Kampala, Uganda and Rockville, Maryland, USA: UBOS and ICF.

[R32] Uganda Bureau of Statistics (UBOS) and ICF International Inc. 2012. *Uganda Demographic and Health Survey 2011*. Kampala, Uganda: UBOS and Calverton, Maryland: ICF International Inc.

[R33] Uganda Bureau of Statistics (UBOS) and Macro International Inc. 2007. *Uganda Demographic and Health Survey 2006*. Calverton, Maryland, USA: UBOS and Macro International Inc.

[R34] USAID & Maternal and Child Health Integrated Program (MCHIP). 2013a.*Introduction to the Quality of Care Surveys. Essential Inventory*. Baltimore, MD: Jhpiego. https://www.mchip.net/sites/default/files/mchipfiles/QoC%20Essential%20Inventory.pdf, accessed 19 February 2020.

[R35] USAID & Maternal and Child Health Integrated Program (MCHIP). 2013b.*Introduction to the Quality of Care Surveys. Labor and Delivery (L&D) Observation Checklist*. Baltimore, MD: Jhpiego. https://www.mchip.net/sites/default/files/mchipfiles/QoC%20LD%20Observation_0.pdf, accessed 19 February 2020.

[R36] WHO. 2011. *WHO Recommendations for Prevention and Treatment of Pre-Eclampsia and Eclampsia*. Geneva: WHO Press. http://apps.who.int/iris/bitstream/10665/44703/1/9789241548335_eng.pdf, accessed 30 August 2020. 23741776

[R37] WHO. 2012a. *Guidelines on Basic Newborn Resuscitation*. Geneva: WHO Press. http://apps.who.int/iris/bitstream/10665/75157/1/9789241503693_eng.pdf, accessed 30 August 2020. 23700652

[R38] WHO. 2012b.*WHO Recommendations for the Prevention and Treatment of Postpartum Haemorrhage*. https://apps.who.int/iris/bitstream/handle/10665/75411/9789241548502_eng.pdf?sequence=1, accessed 30 August 2020. 23586122

[R40] WHO. 2014a.*Every Newborn: an Action Plan to End Preventable Deaths*. World Health Organization. http://www.who.int/maternal_child_adolescent/documents/every-newborn-action-plan/en/, accessed 21 April 2020.

[R39] WHO. 2014b.*Consultation on Improving Measurement of the Quality of Maternal, Newborn and Child Care in Health facilities, WHO*. World Health Organization. http://www.who.int/maternal_child_adolescent/documents/measuring-care-quality/en/, accessed 15 November 2019.

[R41] WHO. 2015a.*Strategies Toward Ending Preventable Maternal Mortality (EPMM)*. World Health Organization. http://www.who.int/reproductivehealth/topics/maternal_perinatal/epmm/en/, accessed 21 April 2020.

[R42] WHO. 2015b. *WHO Safe Childbirth Checklist Implementation Guide*. Geneva: WHO Press. https://apps.who.int/iris/bitstream/handle/10665/199177/9789241549455_eng.pdf?sequence=1, accessed 30 August 2020.

[R43] WHO. 2015c. *Pregnancy, Childbirth, Postpartum and Newborn Care: A Guide for Essential Practice, 3^rd^ Edition*. Geneva: WHO Press. https://apps.who.int/iris/bitstream/handle/10665/249580/9789241549356-eng.pdf?sequence=1, accessed 30 August 2020. 26561684

[R44] WHO. 2016.*Standards for Improving Quality of Maternal and Newborn Care in Health Facilities*. World Health Organization. http://www.who.int/maternal_child_adolescent/documents/improving-maternal-newborn-care-quality/en/, accessed 21 April 2020.

[R45] WHO. 2017a. *WHO Recommendations on Newborn Health: Guidelines Approved by the WHO Guidelines Review Committee*. Geneva: WHO Press, accessed 30 August 2020.

[R46] WHO. 2017b. *Primary Health Care Systems (PRIMASYS): Case Study from Uganda, Abridged Version*. Geneva: World Health Organization. 2017. Licence: CC BY-NC-SA 3.0 IGO, accessed 7 October 2020.

[R47] WHO. 2018. *WHO Recommendations: Intrapartum Care for a Positive Childbirth Experience*. Geneva: World Health Organization. 30070803

[R48] WHO, OECD & International Bank for Reconstruction and Development. 2018.*Delivering Quality Health Services: A Global Imperative for Universal Health Coverage*. World Health Organization. https://apps.who.int/iris/handle/10665/272465, accessed 21 April 2020.

[R50] Zou G. 2004. A modified poisson regression approach to prospective studies with binary data. *American Journal of Epidemiology* 159: 702–6. 15033648 10.1093/aje/kwh090

